# Supplementary material for: Sustainable land management enhances ecological and economic multifunctionality under ambient and future climate
Source: Nat Commun. 2024 Jun 10;15:4930. doi: 10.1038/s41467-024-48830-z (PMC11164979; doi:10.1038/s41467-024-48830-z)
Supplement: Supplementary file 1 — Supplementary Information [file 41467_2024_48830_MOESM1_ESM.pdf]

Sustainable land management enhances ecological and economic multifunctionality under ambient and future climate

Supplementary Material

Table S1 | Overview of datasets used for calculation of ecosystem multifunctionality: Nitrogen surplus was calculated based on data on nitrogen concentration in the soil from 2016 and 2017 and on data on nitrogen deprivation (removal through harvest) from 2016.

	Year									
**Ecosystem function**	**2014**	**2015**	**2016**	**2017**	**2018**	**2019**	**2020**	**2021**	**2022**	**2023**
Yield	x	x	x	x	x	x	x			
Total organic soil carbon	x	x	x	x	x	x	x			
Nitrogen surplus*			x							
Microbial biomass		x	x							
Cellulase activity		x	x	x		x	x			
N-acetylglucosaminidase activity		x	x	x		x	x			
Acid-phosphatase activity		x	x	x		x	x			
Belowground decomposition		x	x							
Aboveground decomposition (microbes)		x	x							
Aboveground decomposition (microbes + fauna)		x	x							
Mesofauna diversity		x	x							
Macrofauna diversity		x	x							
Nematode diversity		x	x							
Flower abundance										x

Table S2 | Effects of land use, climate, and their interaction on ecological ecosystem multifunctionality (equal function and service weighting): General linear mixed-effect regression model table of F and p values (in brackets: numerator and denominator d.f.) of the effect of the two factors land-use type (LUT), and climate type (Climate), and their interaction (LUT:Climate) on ecological ecosystem multifunctionality. For statistical testing, F tests based on ANOVA (two-sided) without adjustments for multiple comparisons were used. Bold values indicate a significant effect of the respective factor or interaction (* p<0.001; ** p<0.01; * p<0.05).**

	LUT			Climate			LUT:Climate		
**Scenario**	**F (4,32)**	**p value**		**F (4,32)**	**p value**		**F (4,32)**	**p value**	
Ecological multifunctionality (equal ecosystem function weighting)	**14.066**	**9.98E-07**	***	**18.698**	**0.0025 32**	**	0.423	0.7906 51	
Ecological multifunctionality (equal ecosystem service weighting)	**46.268**	**7.28E-13**	***	4.707	0.0618 68		2.176	0.0940 77	

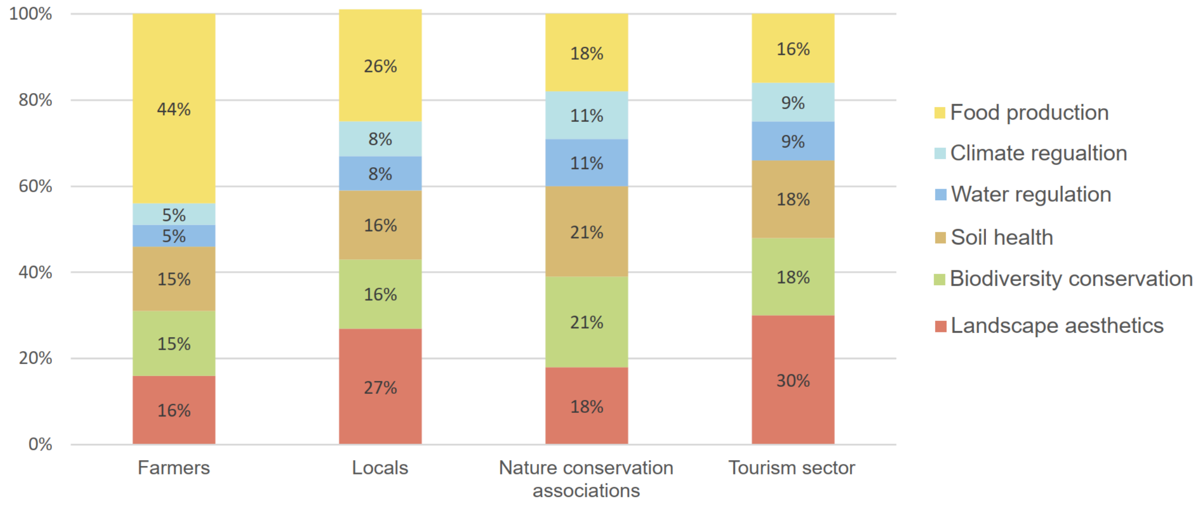

Figure S1 | Weighting of the six ecosystem services considered in this study based on preferences of four different stakeholder groups (preferences derived from Peter et al., 2021[(1)]).

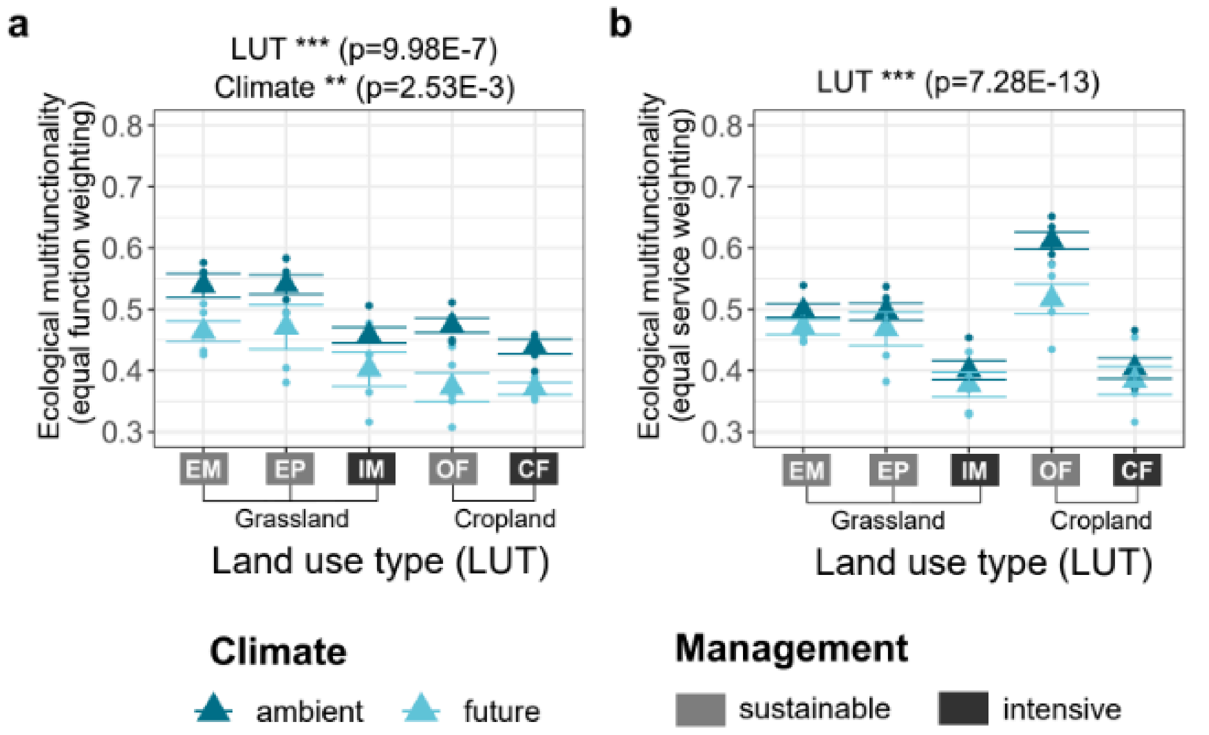

Figure S2 | Ecological ecosystem multifunctionality as affected by five different land-use types (EM: extensive meadow, EP: extensive pasture, IM: intensive meadow, OF: organic farming, CF: conventional farming) for both the ambient and the future climate and for two different weighting scenarios (a: equal ecosystem function weighting, b: equal ecosystem service weighting). Dots indicate the multifunctionality level within the plots of the experiment (5 replicates for each LUT-Climate combination), triangles indicate the mean value of the respective LUT-Climate combination group). For statistical testing, F tests based on ANOVA (two-sided) without adjustments for multiple comparisons were used (numerator df: 4; denominator df: 32). Asterisks indicate a significant effect of the respective factor or interaction (* p<0.001; ** p<0.01; * p<0.05). Error bars represent the standard errors of the mean. Contrary to the weighting according to different stakeholders' preferences, under an equal ecosystem service weighting, ecological ecosystem multifunctionality is not affected by the climate treatment.**

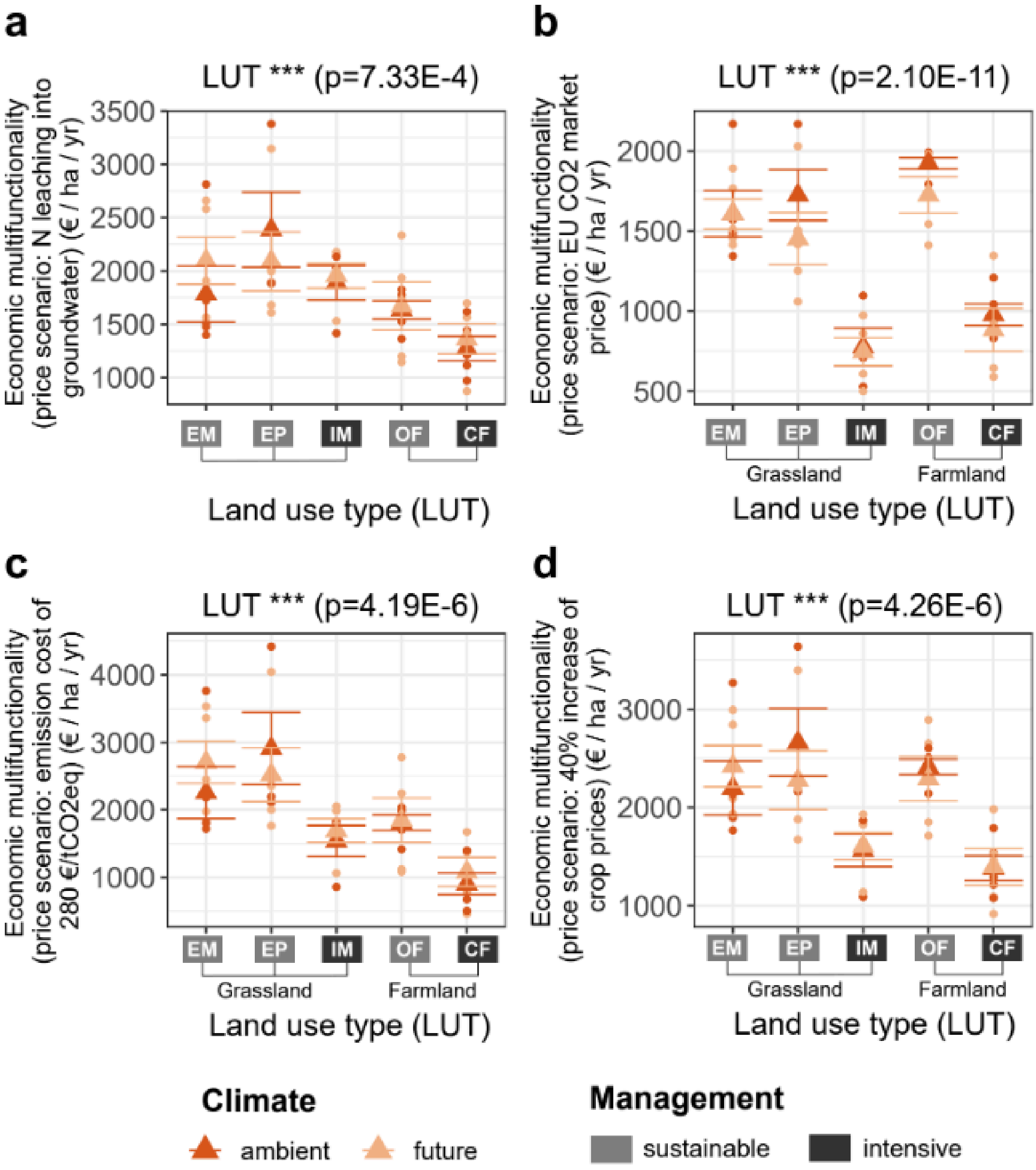

Figure S3 | Economic ecosystem multifunctionality as affected by five different land-use types (EM: extensive meadow, EP: extensive pasture, IM: intensive meadow, OF: organic farming, CF: conventional farming) for both the ambient and the future climate and for four alternative price scenarios (a: social cost of nitrogen leaching into groundwater of 1.90 € / kg; b: market price of CO_2 emission of 90 € / t based on prices within the European Emission Trading System; c: social cost of CO_2 emission of 280 € / t based on Kikstra et al. (2021); d: 40% increase in yield prices). Dots indicate the multifunctionality level within the plots of the experiment (5 replicates for each LUT-Climate combination), triangles indicate the mean value of the respective LUT-Climate combination group). For statistical testing, F tests based on ANOVA (two-sided) without adjustments for multiple comparisons were used (numerator df: 4; denominator df: 32). Asterisks indicate a significant effect of the respective factor or interaction (* p<0.001; ** p<0.01; * p<0.05). Error bars represent the standard errors of the mean. The finding from the original price scenario that economic multifunctionality is increased for sustainable compared to intensive management is robust to the alternative accounting prices of CO_2 emissions and to a 40% increase in crop prices. Only for the alternative accounting price for nitrogen leaching, the effect of higher economic multifunctionality for sustainable management under grassland and cropland vanishes, while the effect of higher economic multifunctionality under grassland remains.**

Note S1 | Ecosystem function responses to different climate and land-use types

Aboveground biomass ('yield') is negatively affected by future climate and, for grassland, significantly higher under intensive management than under sustainable management. Surprisingly, for cropland, yield is higher under sustainable management than under conventional (i.e., intensive) farming. Total organic soil carbon is lower for cropland than for grassland. Nitrogen surplus is significantly higher for the intensively-managed land-use types and in general increased under future climate. Microbial biomass is lower for cropland and shows an interaction effect: future climate increases microbial biomass only in extensive pasture (EP). Effects of land-use type and future climate differ across individual enzymes: cellulase activity is decreased under future climate, while no land-use effect is found. N-acetylglucosaminidase activity is lower for cropland compared to grassland, and not significantly affected by future climate. Acid-phosphatase shows a lower activity only for sustainably-managed cropland. Belowground decomposition rate for grasslands is significantly lower under intensive management, while for cropland no difference was found between the management types. Aboveground (microbial) decomposition rate is lower in croplands than in grasslands. Aboveground (microbial + faunal) decomposition rate is neither affected by climate nor land-use type and intensity. Soil mesofauna diversity is not significantly affected by any treatment, whereas soil macrofauna diversity is lower for cropland compared to grassland. Soil nematode diversity is significantly decreased in intensively-managed grassland compared to all other land-use types.

Table S3 | Effect of land use, climate, and their interaction on ecosystem functions: Linear mixed-effect regression model table of F and p values (in brackets: numerator and denominator d.f.) of the effect of the two factors land-use type (LUT), and climate type (Climate), and their interaction (LUT:Climate) on each individual ecosystem function. For statistical testing, F tests based on ANOVA (two-sided) without adjustments for multiple comparisons were used. Bold values indicate a significant effect of the respective factor or interaction (* p<0.001; ** p<0.01; * p<0.05).**

	LUT			Climate			LUT:Climate		
**Ecosystem function**	**F (4,32)**	**p value**		**F (4,32)**	**p value**		**F (4,32)**	**p value**	
Aboveground biomass	**298.147**	**7.84E-25**	***	**12.015**	**0.008489**	**	0.495	0.739665	
Total organic soil carbon	**11.67**	**5.88E-06**	***	4.336	0.070871		0.241	0.912893	
Nitrogen surplus	**213.658**	**1.36E-22**	***	**5.717**	**0.043793**	*	0.324	0.859821	
Microbial Biomass	**47.251**	**5.48E-13**	***	0	0.987234		1.824	0.148494	
Cellulase activity	**3.049**	**0.030898**	*	2.265	0.170723		0.199	0.93685	
N-acetylgluco-saminidase activity	**17.392**	**1.13E-07**	***	1.37	0.275533		0.338	0.850543	
Acid-phosphatase activity	**10.261**	**1.84E-05**	***	1.241	0.29761		1.319	0.283873	
Belowground decomposition rate	**12.605**	**2.88E-06**	***	5.186	0.052301		2.503	0.061749	
Aboveground decomposition rate (microbes)	**2.946**	**0.035187**	*	**9.069**	0.016778	*	0.991	0.42678	
Aboveground decomposition rate (microbes + fauna)	**3.498**	**0.015346**	*	**4.965**	0.031558	*	1.027	0.405072	
Soil biodiversity (mesofauna)	**11.103**	**9.22E-06**	***	2.599	0.145589		1.07	0.387525	
Soil biodiversity (macrofauna)	**47.238**	**5.50E-13**	***	2.365	0.16265		0.802	0.533246	
Soil biodiversity (nematodes)	**35.524**	**1.14E-12**	***	3.837	0.057135		0.701	0.595913	
Flower abundance	**137.935**	**1.07E-19**	***	**9.564**	**0.014831**	*	**22.04**	**8.15E-09**	***

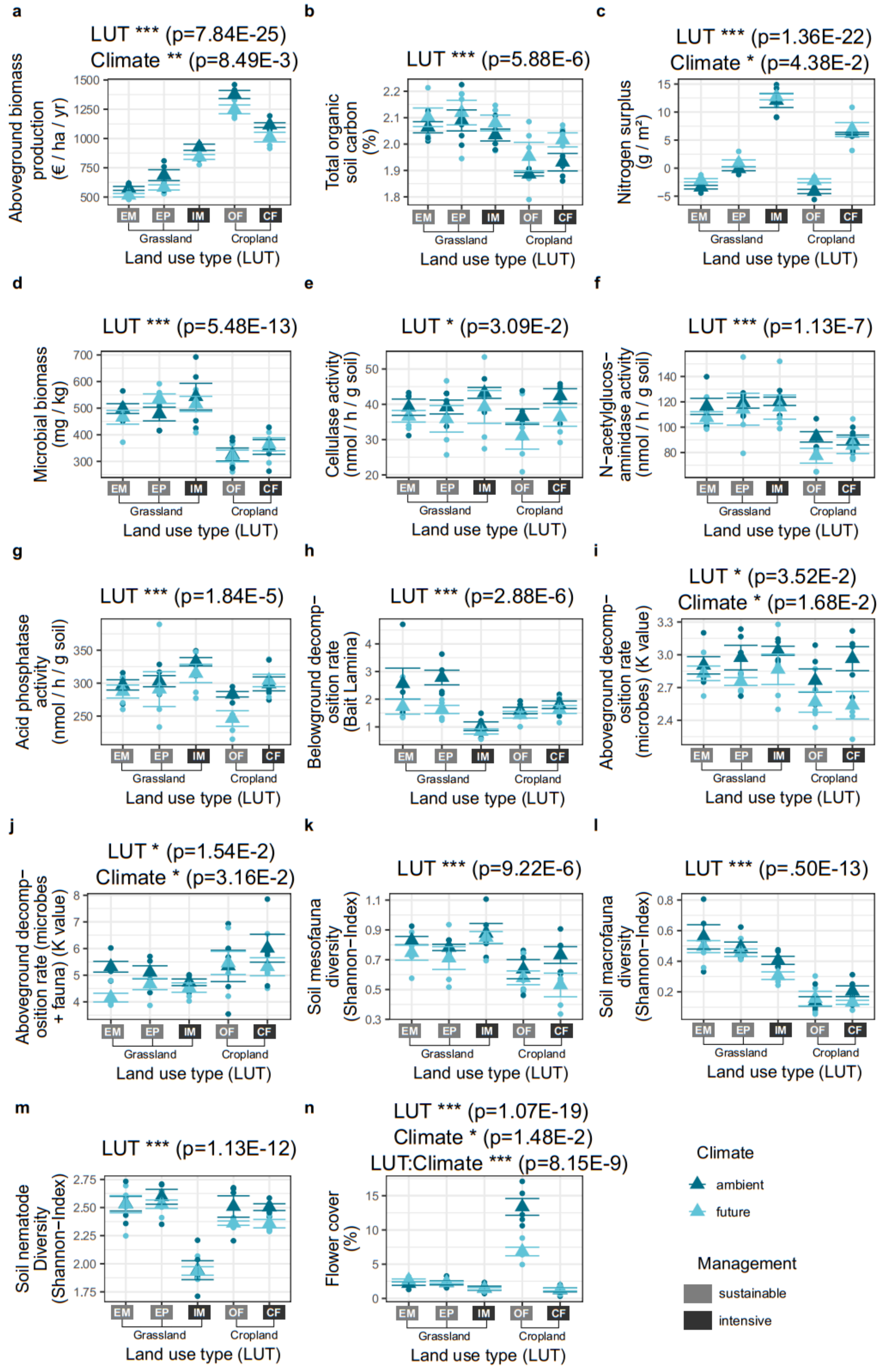

Figure S4 | Individual ecosystem function levels as affected by five different land-use types (EM: extensive meadow, EP: extensive pasture, IM: intensive meadow, OF: organic farming, CF: conventional farming) for both ambient and the future climate. Dots indicate the ecosystem function levels within the plots of the experiment (5 replicates for each LUT-Climate combination), triangles indicate the mean value of the respective LUT-Climate combination group). For statistical testing, F tests based on ANOVA (two-sided) without adjustments for multiple comparisons were used (numerator df: 4; denominator df: 32). Asterisks indicate a significant effect of the respective factor or interaction (* p<0.001; ** p<0.01; * p<0.05). Error bars represent the standard errors of the mean.**

Table S4 | Effect of land use, climate, and their interaction on ecological ecosystem multifunctionality for different stakeholders: Linear mixed-effect regression model table of F and p values (in brackets: numerator and denominator d.f.) of the effect of the two factors land-use type (LUT), climate type (Climate), and their interaction (LUT:Climate) on ecological ecosystem multifunctionality EMF for different weighting scenarios. For statistical testing, F tests based on ANOVA (two-sided) without adjustments for multiple comparisons were used. Bold values indicate a significant effect of the respective factor or interaction (* p<0.001; ** p<0.01; * p<0.05).**

	LUT			Climate			LUT:Climate		
**Weighting**	**F(4,32)**	**p value**		**F(1,8)**	**p value**		**F(4,32) value**	**p value**	
Farmers' preferences	**140.8**	**7.87E-20**	***	**19.05**	**0.002399**	**	**2.96**	**0.034528**	*
Local residents' preferences	**84.73**	**1.47E-16**	***	**15.05**	**0.004679**	**	**6.04**	**0.000978**	***
Environmental conservation agencies' preferences	**29.31**	**2.71E-10**	***	**9.96**	**0.013494**	*	1.69	0.176079	
Tourism sector's preferences	**62.93**	**1.04E-14**	***	**12.34**	**0.007929**	**	**6.61**	**0.00054**	***

Table S5 | Pairwise comparisons of estimated marginal means (EMMs) for the effects of LUT (Land Use Type) on environmental conservation agencies' ecological multifunctionality. The table presents the contrasts, estimates, standard errors (SE), degrees of freedom (df), t-ratios, and p-values. Significant differences between LUT categories are determined using the Tukey adjustment method, whereas bold values indicate a significant effect of the respective factor or interaction (* p<0.001; ** p<0.01; * p<0.05). Negative estimates indicate a decrease in environmental conservation agencies' ecological multifunctionality compared to the reference category. Positive estimates indicate an increase in environmental conservation agencies' ecological multifunctionality compared to the reference category.**

**contrast**	**estimate**	**SE**	**df**	**t.ratio**	**p.value**	
EM - EP	-0.001	0.014	36	-0.045	0.999999	
**EM - IM**	**0.07**	**0.014**	**36**	**4.885**	**0.000197**	***
EM - OF	-0.033	0.014	36	-2.331	0.158732	
**EM - CF**	**0.092**	**0.014**	**36**	**6.404**	**1.94E-06**	***
**EP - IM**	**0.071**	**0.014**	**36**	**4.93**	**0.000172**	***
EP - OF	-0.033	0.014	36	-2.286	0.172954	
**EP - CF**	**0.093**	**0.014**	**36**	**6.449**	**1.69E-06**	***
**IM - OF**	**-0.104**	**0.014**	**36**	**-7.216**	**1.67E-07**	***
IM - CF	0.022	0.014	36	1.519	0.55734	
**OF - CF**	**0.125**	**0.014**	**36**	**8.735**	**1.99E-09**	***

Table S6 | Effect of land use, climate, and their interaction on economic ecosystem multifunctionality. Linear mixed-effect regression model table of F and p values (in brackets: numerator and denominator d.f.) of the effect of the two factors land-use type (LUT), climate type, and their interaction on economic ecosystem multifunctionality value (enEMF) and on farmers' economic ecosystem multifunctionality (enEMF (farmers)). For statistical testing, F tests based on ANOVA (two-sided) without adjustments for multiple comparisons were used. Bold values indicate a significant effect of the respective factor or interaction (* p<0.001; ** p<0.01; * p<0.05).**

	**LUT**			**Climate**			**LUT:Climate**		
**Value**	**F(4,32)**	**p value**		**F(1,8)**	**p value**		**F(4,32)**	**p value**	
Economic multifunctionality enEMF	**14.94**	**7.06E-07**	***	0	0.997391		0.56	0.690508	
Farmers' economic multifunctionality enEMF (farmers)	**153.03**	**2.23E-20**	***	**34.559**	**0.000371**	***	0.33	0.856238	

Table S7 | Pairwise comparisons of estimated marginal means (EMMs) for the effects of LUT (Land Use Type) on economic multifunctionality. The table presents the contrasts, estimates, standard errors (SE), degrees of freedom (df), t-ratios, and p-values. Significant differences between LUT categories are determined using the Tukey adjustment method, whereas bold values indicate a significant effect of the respective factor or interaction (* p<0.001; ** p<0.01; * p<0.05). Negative estimates indicate a decrease in economic multifunctionality compared to the reference category. Positive estimates indicate an increase in economic multifunctionality compared to the reference category.**

**contrast**	**estimate**	**SE**	**df**	**t.ratio**	**p.value**	
EM - EP	-95.827	195.329	34.414	-0.491	0.987728	
**EM - IM**	**862.719**	**195.329**	**34.414**	**4.417**	**0.000849**	***
EM - OF	263.569	189.165	34.016	1.393	0.635979	
**EM - CF**	**1126.403**	**189.165**	**34.016**	**5.955**	**9.37E-06**	***
**EP - IM**	**958.547**	**201.454**	**34.952**	**4.758**	**0.000305**	***
EP - OF	359.396	195.329	34.414	1.84	0.368114	
**EP - CF**	**1222.23**	**195.329**	**34.414**	**6.257**	**3.64E-06**	***
**IM - OF**	**-599.151**	**195.329**	**34.414**	**-3.067**	**0.031927**	*
IM - CF	263.683	195.329	34.414	1.35	0.66266	
**OF - CF**	**862.834**	**189.165**	**34.016**	**4.561**	**0.000571**	***

Table S8 | Pairwise comparisons of estimated marginal means (EMMs) for the effects of LUT (Land Use Type) on farmers' economic multifunctionality. The table presents the contrasts, estimates, standard errors (SE), degrees of freedom (df), t-ratios, and p-values. Significant differences between LUT categories are determined using the Tukey adjustment method, whereas bold values indicate a significant effect of the respective factor or interaction (* p<0.001; ** p<0.01; * p<0.05). Negative estimates indicate a decrease in farmers' economic multifunctionality compared to the reference category. Positive estimates indicate an increase in farmers' economic multifunctionality compared to the reference category.**

contrast	estimate	SE	df	t.ratio	p.value	
**EM - EP**	**-89.263**	**27.212**	**32**	**-3.280**	**0.019705**	*
**EM - IM**	**-298.433**	**27.212**	**32**	**-10.967**	**2.24E-11**	***
**EM - OF**	**-596.355**	**27.212**	**32**	**-21.915**	**1.00E-13**	***
**EM - CF**	**-387.669**	**27.212**	**32**	**-14.246**	**1.23E-13**	***
**EP - IM**	**-209.170**	**27.212**	**32**	**-7.687**	**8.94E-08**	***
**EP - OF**	**-507.092**	**27.212**	**32**	**-18.635**	**1.00E-13**	***
**EP - CF**	**-298.406**	**27.212**	**32**	**-10.966**	**2.24E-11**	***
**IM - OF**	**-297.922**	**27.212**	**32**	**-10.948**	**2.34E-11**	***
**IM - CF**	**-89.235**	**27.212**	**32**	**-3.279**	**0.019755**	*
**OF - CF**	**208.686**	**27.212**	**32**	**7.669**	**9.39E-08**	***

Table S9 | Effect of land use, climate, and their interaction on economic ecosystem multifunctionality for alternative price scenarios. Linear mixed-effect regression model table of F and p values (in brackets: numerator and denominator d.f.) of the effect of the two factors land-use type (LUT), climate type (Climate), and their interaction (LUT:Climate) on ecological multifunctionality for different weighting scenarios. For statistical testing, F tests based on ANOVA (two-sided) without adjustments for multiple comparisons were used. Bold values indicate a significant effect of the respective factor or interaction (* p<0.001; ** p<0.01; * p<0.05).**

	**LUT**			**Climate**			**LUT:Climate**		
**Scenario**	**F (4,32)**	**p value**		**F (4,32)**	**p value**		**F (4,32)**	**p value**	
Economic multifunctionality (alternative price scenario, CO2 price of 90 €/t, according to ETS)	**37.589**	**2.10E-11**	***	1.548	0.247826		0.545	0.704028	
Economic multifunctionality (alternative price scenario, N leaching social cost of 1.9 € / kg, according to Federal German Environment Agency)	**6.385**	**0.000733**	***	0.083	0.780477		0.619	0.652407	
Economic multifunctionality (alternative price scenario, CO2 price of 280 €/t, according to Kikstra et al., 2021)	**12.376**	**4.19E-06**	***	0.152	0.706977		0.574	0.683679	
Economic multifunctionality (alternative price scenario, 40% increase in yield prices)	**12.348**	**4.26E-06**	***	0.052	0.824514		0.615	0.655326	

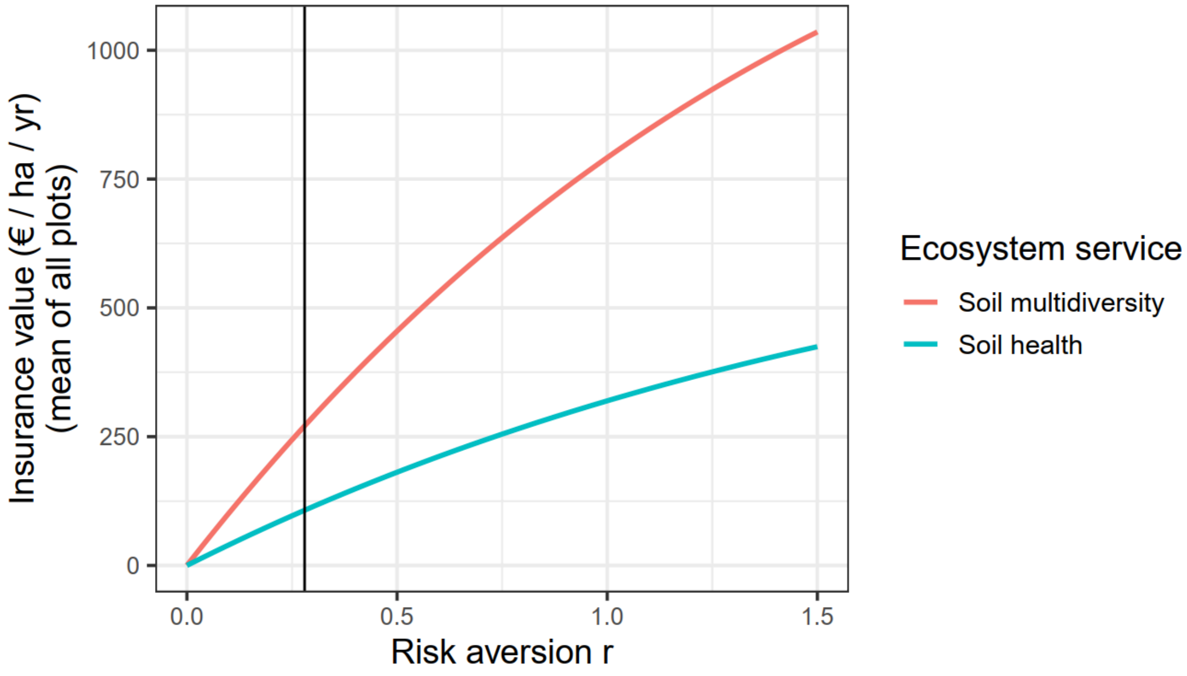

Figure S5 | Insurance value (mean of all plots) as function of risk aversion for both biodiversity and soil health. Vertical bar indicates the value of risk aversion r that was chosen for this study (0.28 as the relative risk aversion of a slightly risk averse farmer according to Tevenart et al., 2021).

Table S10 | Producer prices used for the conversion of the aboveground biomass yield unit from dry biomass into monetary value of the yield with information regarding source and conversion process.

Crop	Price	Source	Notes
Winter wheat organic (*Triticum aestivum*)	254.09 € / t	Agrarmarkt Austria. (2020).	Mean over 5 years (2015/16 – 2019/20).
Winter barley organic (*Hordeum vulgare*)	221.14 € / t		
Horse bean organic (*Vicia faba*)	401.16 € / t		
Straw	102.40 € / t	Proplanta Informationszentrum für Landwirtschaft. (2021).	Mean over 6 years (2014-2019), for every year: mean from calendar week 12,24,36 and 48 was calculated.
Hay	146.30 € / t		
Winter wheat conventional (*Triticum aestivum*)	163.30 € / t	Bundesministerium für Ernährung und Landwirtschaft. (2020).	Mean over 6 years (2014 – 2019). As winter barley is mainly cultivated as fodder barley (Thüringer Landesanstalt für Landwirtschaft, 2015), the price for fodder barley is used for the converting.
Fodder barley conventional (*Hordeum vulgare*)	147.00 € / t		
Rape (*Brassica napus*)	351.80 € / t		

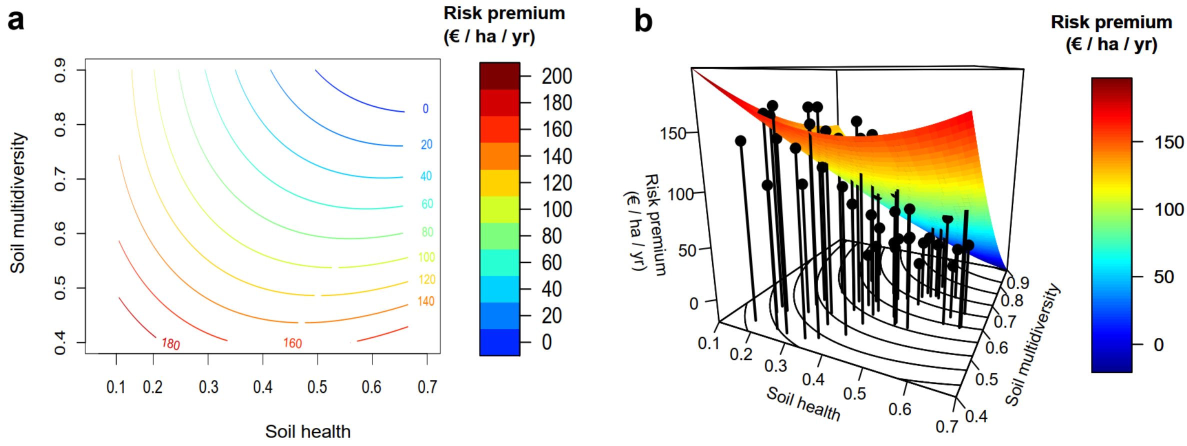

Figure S6 | Risk premium as function of biodiversity and soil health (normalised levels) for a risk aversion value of r = 0.28 as the relative risk aversion of a slightly risk averse farmer according to Tevenart et al. (2021): response surface used for the calculation of the insurance value of biodiversity and soil health in two (a) and three dimensions (b).

References

Agrarmarkt Austria. 2020. Bio-Erzeugerpreise endgültig. https://www.ama.at/getattachment/cc664e59-0761-4585-a35a-b0ff1b5d9926/Bio_Erzeugerpreise_2019_2020.pdf. Retrieved 29-06-2021

Bundesministerium für Ernährung und Landwirtschaft. 2020. Statistisches Jahrbuch über Ernährung, Landwirtschaft und Forsten der Bundesrepublik Deutschland 2020, p. 216. https://www.bmel-statistik.de/fileadmin/SITE_MASTER/content/Jahrbuch/Agrarstatistisches-Jahrbuch-2020.pdf. Retrieved 29-06-2021

Kikstra, J. S., Waidelich, P., Rising, J., Yumashev, D., Hope, C., & Brierley, C. M. (2021). The social cost of carbon dioxide under climate-economy feedbacks and temperature variability. *Environmental Research Letters*, *16*(9), 094037. https://doi.org/10.1088/1748-9326/ac1d0b

Tevenart, C., Brunette, M. (2021). Role of Farmers' Risk and Ambiguity Preferences on Fertilization Decisions: An Experiment. *Sustainability*, *13*, 9802. https://doi.org/10.3390/su13179802

Thüringer Landesanstalt für Landwirtschaft. 2015. „Leitlinie zur effizienten und umweltverträglichen Erzeugung von Wintergerste" http://www.tll.de/ainfo/pdf/ll_wg.pdf (Seite 4). Retrieved 29-06-2021

Peter, S., Le Provost, G., Mehring, M., Müller, T., & Manning, P. (2022). Cultural worldviews consistently explain bundles of ecosystem service prioritisation across rural Germany. *People and Nature*, *4*(1), 218–230. https://doi.org/10.1002/pan3.10277

Proplanta Informationszentrum für Landwirtschaft. 2021. Aktuelle Strohpreise und Heupreise. https://www.proplanta.de/markt-und-preis/agrarmarkt-berichte/aktuelle-strohpreise-und-heupreise/agrar_marktnews_themen.php?SITEID=1148888702&ROalAk=377&LaZ=25&LsZ=75&ROalAk=378&EgSa=888&template_id=943916431. Retrieved 29-06-2021
