# Supplementary material for: Sustainable land management enhances ecological and economic multifunctionality under ambient and future climate
Source: Nat Commun. 2024 Jun 10;15:4930. doi: 10.1038/s41467-024-48830-z (PMC11164979; doi:10.1038/s41467-024-48830-z)
Supplement: Supplementary file 2 — Reporting Summary [file 41467_2024_48830_MOESM2_ESM.pdf]

nature portfolio

Corresponding author(s): Prof. Dr. Nico Eisenhauer

Last updated by author(s): Apr 25, 2024

Reporting Summary

Nature Portfolio wishes to improve the reproducibility of the work that we publish. This form provides structure for consistency and transparency in reporting. For further information on Nature Portfolio policies, see our Editorial Policies and the Editorial Policy Checklist.

Statistics

For all statistical analyses, confirm that the following items are present in the figure legend, table legend, main text, or Methods section.

n/a	Confirmed	
☐	☒	The exact sample size (*n*) for each experimental group/condition, given as a discrete number and unit of measurement
☐	☒	A statement on whether measurements were taken from distinct samples or whether the same sample was measured repeatedly
☐	☒	The statistical test(s) used AND whether they are one- or two-sided<br>*Only common tests should be described solely by name; describe more complex techniques in the Methods section.*
☒	☐	A description of all covariates tested
☐	☒	A description of any assumptions or corrections, such as tests of normality and adjustment for multiple comparisons
☐	☒	A full description of the statistical parameters including central tendency (e.g. means) or other basic estimates (e.g. regression coefficient) AND variation (e.g. standard deviation) or associated estimates of uncertainty (e.g. confidence intervals)
☐	☒	For null hypothesis testing, the test statistic (e.g. *F*, *t*, *r*) with confidence intervals, effect sizes, degrees of freedom and *P* value noted<br>*Give P values as exact values whenever suitable.*
☒	☐	For Bayesian analysis, information on the choice of priors and Markov chain Monte Carlo settings
☐	☒	For hierarchical and complex designs, identification of the appropriate level for tests and full reporting of outcomes
☐	☒	Estimates of effect sizes (e.g. Cohen's *d*, Pearson's *r*), indicating how they were calculated

Our web collection on statistics for biologists contains articles on many of the points above.

Software and code

Policy information about availability of computer code

Data collection	R (v.4.0.4; R Core Team, 2021)<br>Code is available under XXXX (CREATIVE LIVENCE??? --> diesen Text auch in Manuskript einfügen)<br><br>Python (v.3.7; Python Software Foundation, 2018)<br>Code available under https://doi.org/10.5281/zenodo.10901696. There are no restrictions.
Data analysis	Libraries used for Python:<br>PIL (VERSION)<br>pandas (VERSION)<br>os (VERSION)<br><br>Packages used for R:<br>ggplot2 (v.3.3.5)<br>ggpubr (v.0.4.0)<br>lme4 (v.1.1.27)<br>lmerTest (v.3.1.3)<br>plot3D (v.1.4)<br>car (v.3.0.10)<br>multcomp (v.1.4.17)

multcompView (v.0.1.8)
emmeans (v.1.6.1)
stringr (v.1.4.0)
dplyr (v.1.0.6)
tibble (v.3.1.2)
lsmeans (v.2.30.0)
DescTools (v.0.99.44)

For manuscripts utilizing custom algorithms or software that are central to the research but not yet described in published literature, software must be made available to editors and reviewers. We strongly encourage code deposition in a community repository (e.g. GitHub). See the Nature Portfolio guidelines for submitting code & software for further information.

Data

Policy information about availability of data

All manuscripts must include a data availability statement. This statement should provide the following information, where applicable:

- Accession codes, unique identifiers, or web links for publicly available datasets
- A description of any restrictions on data availability
- For clinical datasets or third party data, please ensure that the statement adheres to our policy

Code for data cleaning and analysis has been deposited in the Zenodo database under Creative Commons Attribution licence (refs 96,106).

Research involving human participants, their data, or biological material

Policy information about studies with human participants or human data. See also policy information about sex, gender (identity/presentation), and sexual orientation and race, ethnicity and racism.

Reporting on sex and gender	not applicable
Reporting on race, ethnicity, or other socially relevant groupings	not applicable
Population characteristics	not applicable
Recruitment	not applicable
Ethics oversight	not applicable

Note that full information on the approval of the study protocol must also be provided in the manuscript.

Field-specific reporting

Please select the one below that is the best fit for your research. If you are not sure, read the appropriate sections before making your selection.

☐ Life sciences ☐ Behavioural & social sciences ☒ Ecological, evolutionary & environmental sciences

For a reference copy of the document with all sections, see nature.com/documents/nr-reporting-summary-flat.pdf

Life sciences study design

All studies must disclose on these points even when the disclosure is negative.

Sample size	n = 50 experimental plots.
Data exclusions	No data excluded.
Replication	The GCEF was designed for a simultaneous manipulation of land-use type (5 types: 2 cropland and 3 grassland types) and climate (ambient and future), using a fully randomised split plot experimental design that allows full-factorial combination of the climate and land-use types with 50 plots of approximately 400 m^2 each (5 replicates for each land-use type-climate treatment combination) (ref 33)The.
Randomization	The experiment has a fully randomised split plot experimental design.
Blinding	Blinding was not possible due to field sampling campaigns and obvious differences among experimental treatments.

Behavioural & social sciences study design

All studies must disclose on these points even when the disclosure is negative.

Study description	*Briefly describe the study type including whether data are quantitative, qualitative, or mixed-methods (e.g. qualitative cross-sectional, quantitative experimental, mixed-methods case study).*
Research sample	*State the research sample (e.g. Harvard university undergraduates, villagers in rural India) and provide relevant demographic information (e.g. age, sex) and indicate whether the sample is representative. Provide a rationale for the study sample chosen. For studies involving existing datasets, please describe the dataset and source.*
Sampling strategy	*Describe the sampling procedure (e.g. random, snowball, stratified, convenience). Describe the statistical methods that were used to predetermine sample size OR if no sample-size calculation was performed, describe how sample sizes were chosen and provide a rationale for why these sample sizes are sufficient. For qualitative data, please indicate whether data saturation was considered, and what criteria were used to decide that no further sampling was needed.*
Data collection	*Provide details about the data collection procedure, including the instruments or devices used to record the data (e.g. pen and paper, computer, eye tracker, video or audio equipment) whether anyone was present besides the participant(s) and the researcher, and whether the researcher was blind to experimental condition and/or the study hypothesis during data collection.*
Timing	*Indicate the start and stop dates of data collection. If there is a gap between collection periods, state the dates for each sample cohort.*
Data exclusions	*If no data were excluded from the analyses, state so OR if data were excluded, provide the exact number of exclusions and the rationale behind them, indicating whether exclusion criteria were pre-established.*
Non-participation	*State how many participants dropped out/declined participation and the reason(s) given OR provide response rate OR state that no participants dropped out/declined participation.*
Randomization	*If participants were not allocated into experimental groups, state so OR describe how participants were allocated to groups, and if allocation was not random, describe how covariates were controlled.*

Ecological, evolutionary & environmental sciences study design

All studies must disclose on these points even when the disclosure is negative.

Study description	We analysed data from the years 2014-2023 from the Global Change Experimental Facility (GCEF) in Saxony-Anhalt, Germany, a large field experiment with orthogonal manipulation of climate (ambient and future climate type) and land use (five different land-use types: three grassland and two farmland types either sustainably managed (no application of mineral nitrogen fertiliser and pesticides) or intensively managed (application of mineral nitrogen fertiliser and pesticides), see Table 1) within 50 plots of approximately 400 m² each (Schädler et al., 2019). The 50 plots were grouped in 10 mainplots, whereas each mainplot contains all five land use types, and five mainplots are managed under ambient climate and five mainplots under future climate (i.e., 5 replications for each combination of land use and climate type). We measured the levels of 14 ecosystem functions approximating six ecosystem services across over seven years after establishment of the treatments
Research sample	On 50 experimental plots, data on 14 different ecosystem services was assessed over seven years, sampling varies according to each ecosystem function (see Methods). The sample size and composition at the GCEF were determined to ensure robust statistical power and representativeness of the broader population of interest. The selection of 50 plots, each approximately 400 m² in size, was based on considerations of experimental feasibility, logistical constraints, and statistical requirements.<br><br>The decision to organize the plots into 10 mainplots, with each mainplot containing all five land-use types, was made to facilitate the implementation of the orthogonal manipulation of climate and land use. This design allows for the replication of treatments across different combinations of land use and climate types, thereby enhancing the validity and generalizability of the findings.<br><br>Additionally, the duration of the study, spanning over seven years, was chosen to capture the long-term dynamics of ecosystem functioning and assess potential temporal trends or responses to the experimental treatments.<br><br>The research sample at the GCEF represents agricultural landscapes typical of the temperate region in central Germany. The findings from this study are intended to contribute to our understanding of how global change drivers, such as climate change and land-use intensification, may impact ecosystem services in similar ecosystems worldwide.
Sampling strategy	Measurements in this study were taken from distinct samples representing unique observations in the study area.<br><br>The sampling procedure at the Global Change Experimental Facility (GCEF) involved systematic selection of plots within the experimental site. The 50 plots, each approximately 400 m² in size, were established to ensure spatial representativeness across the study area. Within each mainplot, the five different land-use types were randomly assigned to minimize potential bias and ensure treatment independence.<br><br>Data collection on ecosystem functions was conducted through regular monitoring and measurements taken at predetermined

intervals over the course of seven years following the establishment of the experimental treatments. Detailed protocols for data collection and measurement techniques are provided in the methodology section.

The sample size of 50 plots was determined based on a combination of statistical considerations and practical constraints. While no formal sample size calculation was performed, the chosen sample size was deemed sufficient to detect meaningful differences in ecosystem functions across the different treatments and ensure robust statistical power. This decision was informed by previous research conducted at similar experimental sites and discussions among the research team regarding the feasibility and logistical constraints of the study.

Additionally, the duration of the study period, spanning over seven years, was considered adequate to capture temporal variations in ecosystem functioning and assess the long-term effects of the experimental treatments.

Data collection

Measurements in this study were taken from distinct samples representing unique observations in the study area. During the years 2014 to 2020, plots were harvested with a combine harvester. Yield biomass (dt / ha, for cropland differentiated into grain and straw yields) was measured after air drying which left the biomass with residual moisture of 14% (barley / wheat grains) and 9% (rape grains). Depending on the annual environmental conditions, for grassland, harvesting occurred up to four times per year. For the total productivity over the year, yields of all harvests are summed up for each plot. Data on yield (machine harvest) was collected and recoreded by Martin Schädler and Ines Merbach.

For extensive pasture, machine harvest was not practical, as plots were grazed with sheep. Instead, harvesting was done manually right above the soil (for each plot, four subsamples were taken and averaged). Here, total yield also considers the grazing uptakes of the sheep, measured as the difference between the biomass in four subsamples in sheep-excluding cages and the four subsamples in the sheep area. Data on yield (manual) was collected and recorded by Harald Auge, Martin Schädler, and Ines Merbach).

Soil samples were taken 2015 and 2016 in the beginning of September from the topsoil layer (0-15 cm) by Thomas Reitz . Twenty drillings were collected per plot, pooled together, and sieved at 2 mm. Total organic soil carbon contents (% of dry soil) were determined in duplicate via dry combustion using a Vario EL III C/H/N analyser (Elementar, Hanau, Germany) according to Breitkreuz et al. (2021). Reporting was done by Thomas Reitz and Evgenia Blagodatskaya.

All measurements in the following were conducted in water baths at 20°C in an air-conditioned laboratory at iDiv using an automated O2-microcompensation system (Scheu, 1992). Before the start of measurements, samples were kept at 20°C for 5 days to adapt the soil microbial community to a constant and standardised temperature. Soil microbial biomass C (MBC) was measured by substrateinduced respiration, i.e., the respiratory response of microorganisms to glucose (Anderson & Domsch, 1978). To saturate catabolic microbial enzymes, 8 mg glucose g-1 soil dry weight was added as aqueous solution to the soil samples. The mean of the three lowest hourly measurements within the first 10 h (excluding the first 2 h) was taken as the maximum initial respiratory response (MIRR)—a period where microbial growth has not started. Microbial biomass (!g C g-1 dry soil) was calculated as 38 × MIRR (!l O2 g-1 dry soil), following the procedure of Beck et al. (1997). Reporting was done by Nico Eisenhauer.

Enzymatic activities (nmol / h / g dry soil) were determined in years 2015, 2016, 2017, 2019 and 2020 using 4-methylumbelliferone (MUF)-labelled substrates according to Francioli et al. (2016). Three enzymes (cellulase, N-acetylglucosaminidase, acid-phosphatase) that are ubiquitous in most organisms and represent the carbon, nitrogen, and phosphorus cycles were measured as indicator for the rate at which microbes can decompose and process organic matter to provide nutrients that are accessible to plants (phosphorous, nitrogen). For the measurements, individual black 96-well microplates were set up for each soil sample. These plates included enzyme-specific substrates, MUF dilutions (at 1.25 and 2.5 !M) to calculate quench and extinction coefficients, as well as controls for substrate and soil suspension. Approximately 250 mg of fresh soil sample was then suspended in 50 ml of acetate buffer (50 mM, pH 5) for analysis. To disrupt soil aggregates, the soil suspensions were sonicated for 5 min, transferred to the prepared microplates, and incubated at 25°C for 60 min. The addition of 30 !l of 1 M NaOH solution stopped the enzymatic reactions. Fluorescence measurements were conducted using an Infinite 200 PRO plate reader (Tecan Group, Männedorf, Switzerland) with excitation at 360 nm and emission at 465 nm. Enzyme activities were reported as the turnover rate of the substrate in nmol per gram of dry soil per hour. Reporting was done by Thomas Reitz.

Belowground decomposition rate that is closely linked to nutrient cycles and includes effects of soil meso- and macrofauna was measured 3-weekly from 2015 to 2016 using bait lamina stripes (average of 6 bait lamina stripes within one plot that remained in the soil for 3 weeks; see Siebert et al., 2019 for details). Data collection and reporting was done by Julia Siebert.

Aboveground decomposition rate (microbes / microbes + fauna) was measured using litterbags (0.02mm / 5mm mesh size) left on soil for two (summer and spring) or 4 months (winter) with a total of 7 measurement periods, starting in April 2015 (see Yin et al. (2019a) for methodological details). Soil mineral nitrogen (NH4+ and NO3-) content (mg / kg dry soil) was measured in 3-weekly resolution from 2015 to 2017 according to Breitkreuz et al. (2021). Data collection and reporting was done by Rui Yin.

Mineral nitrogen deprivation (the removal of nutrients from the soil, in kg N / ha) was measured over the years 2016 to 2019 through an elemental analysis of the harvested plant biomass (for organic and conventional farming only for the machine harvest (cut 10 cm above soil), for intensive and extensive meadow also for the manual harvest (cut 3 cm above the soil surface), for extensive pasture only for the manual harvest). Plant material (dried at 70°C for 48 hours) was shredded and homogenised, a subsample was milled to a fine powder, and appr. 10 mg of the finely milled plant material were weighted with an analytical microbalance (Cubis MSA 3.6P, Sartorius AG, Göttingen, Germany) into tin capsules and measured with an elemental analyser (Vario EL cube, Elementar Analysensysteme GmbH, Langenselbold, Germany). Nitrogen stocks were calculated based on data on yield (dry biomass). Data collection and reporting was done by Christiane Roscher and Stan Harpole.

Soil nematode biodiversity was measured in 2015 and 2016 (two measurements per year, in both spring and autumn). Seven soil subsamples per plot were taken using a steel corer (1 cm diameter; 15 cm depth), homogenised, sieved at 2 mm, and stored at 4°C. Nematodes were extracted with a modified Baermann method (Ruess 1995), whereas for each plot approximately 25 g of soil was

transferred to plastic vessels with a milk filter and a fine gauze (200 µm) at the bottom and placed in water-filled funnels. To ensure soil sample saturation and to ensure a connected water column throughout the sample and the funnel that allowed nematode migration from the soil through the milk filter and the gauze into the funnel, more water was added. After migration, nematodes gravitationally settled at the bottom of a closed tube connected to the funnel, and after 72 h at 20°C, were transferred to a 4% formaldehyde solution, and were counted at 100 × magnification by using a Leica DMI 4000B light microscope. Identification was conducted at 400 × magnification. In order to identify the specimens, sediment material from the bottom of each sample was collected using a 2 ml plastic pipette. The collected sediment was then examined on temporary microscope slides. A minimum of 100 well-preserved specimens, chosen at random from the sample (if available), were identified up to the genus level for adults and most juveniles, or at the family level for juveniles, using the classification method described by Bongers (1988). Subsequently, the nematode taxa were categorised into trophic groups, including bacteria, fungal, and plant feeders, omnivores, and predators, and nematode Shannon diversity index was calculated using the R package 'vegan' for both spring and autumn sample, and averaged for each year, see Siebert et al. (2020) for further methodological details. Data collection and reporting was done by Marcel Ciobanu and Nico Eisenhauer.

Soil meso- and macrofauna diversity was measured in year 2015 and 2016 (two measurements per year (spring and autumn). During each sampling event, three soil core samples (with a diameter of 6 cm and depth of 5 cm) were collected per plot to extract mesofauna, primarily consisting of Collembola and Acari. The Macfadyen high-gradient extractor (Macfadyen, 1961) was employed for this purpose. Collembolans were identified up to the family level, while Acari were identified up to the order level, utilising a VHXDigital microscope. For macrofauna, two soil cores (with a diameter of 16 cm and depth of 5 cm) were taken per plot. The Kempson extraction method (Kempson et al., 1963) was employed, which involved gradually increasing the temperature over a span of 10 days. Macrofauna at the family level (Staphylinidae, Carabidae, and Formicidae), order level (Diptera, Araneae, Isopoda, Haplotaxida, Julida, and Psocoptera), or class level (Chilopoda, Araneae, Symphyla, and Gastropoda) were identified and Shannon index was calculated accordingly (separate for meso- and macrofauna, and for the spring and autumn samples), and averaged for each year, for further methodological details see Yin et al. (2019b). Data was collected and reported by Rui Yin.

Timing and spatial scale

Yield was measured etween years 2014 and 2020 during harvest between May and August. These time points were chosen to align to common agricultural practice in the area of the experiment.

Total organic soil carbon was measured between years 2014 and 2020, in the beginning of September. September was selected to coincide with the end of the growing season.

Microbial biomass was measured in years 2015 and 2016, in the beginning of September. September was selected to coincide with the end of the growing season.

Enzymatic activity was measured in years 2015, 2016, 2017, 2019, 2020, in the beginning of September. September was selected to coincide with the end of the growing season.

Belowground decomposition rate was measured 3-weekly from 2015 to 2016 . This frequency was chosen to capture the rapid turnover of organic matter and to assess short-term decomposition dynamics.

Aboveground decomposition rate (microbes / microbes + fauna) was measured using litterbags left on soil for two (summer and spring) or 4 months (winter) with a total of 7 measurement periods, starting in April 2015 until March 2017. This approach allowed for the assessment of decomposition rates across different seasons and under varying environmental conditions.

Soil mineral nitrogen (NH4+ and NO3-) content was measured in 3-weekly resolution from 2015 to 2017. This frequent sampling frequency was chosen to capture the temporal variability in soil nitrogen availability and to assess the effects of management practices on nutrient cycling.

Mineral nitrogen deprivation was measured over the years 2016 to 2019. This period was selected to assess the long-term effects of nitrogen fertilization on soil nitrogen dynamics and plant nutrient uptake.

Soil nematode biodiversity was measured in 2015 and 2016 (two measurements per year, in both spring and autumn). These seasons were chosen to capture the seasonal fluctuations in nematode abundance and community composition.

Soil meso- and macrofauna diversity was measured in year 2015 and 2016 (two measurements per year (spring and autumn). These seasons were chosen to capture the seasonal fluctuations in nematode abundance and community composition.

Flower cover was measured in 2023 (beginning of June). This timing was chosen to coincide with the peak of flowering activity.

Data exclusions

No data was excluded from the analysis.

Reproducibility

The reproducibility of the experimental findings was ensured through precise documentation of the experimental setup, standardized protocols for data collection, and transparency in reporting results. The unique experimental setup at the Global Change Experimental Facility (GCEF) in Saxony-Anhalt, Germany, was designed to facilitate reproducibility and robustness of the findings.

For each experiment conducted within the GCEF, rigorous measures were taken to verify the reproducibility of the results. Standardized protocols and procedures were followed consistently across all experiments to minimize variability and ensure consistency in data collection methods.

It is noted that all attempts to repeat the experiments were successful, and the findings reported in this study are reproducible under

similar experimental conditions. No instances of failed attempts to repeat the experiments were encountered during the course of the study.

Randomization | Samples within the Global Change Experimental Facility (GCEF) were allocated into experimental groups using randomized procedures. Plots were arranged randomly to ensure impartial allocation of treatments, minimize biases, and control for potential sources of variation. Randomization was rigorously implemented to enhance the internal validity of the study and ensure the integrity of the experimental design. No instances of non-random allocation or bias in treatment assignment were encountered.

Blinding | Blinding was not possible due to field sampling campaigns and obvious differences among experimental treatments.

Did the study involve field work? ☒ Yes ☐ No

Field work, collection and transport

Field conditions | Field conditions, including temperature, precipitation, soil moisture, and light availability, varied over the study period at the Global Change Experimental Facility (GCEF) in Saxony-Anhalt, Germany. These environmental factors influenced ecosystem processes and species interactions within the experimental plots.

Location | 51° 23’ 30N, 11° 52’ 49E, 116 m a.s.l.

Access & import/export | Access to the study site at the Global Change Experimental Facility (GCEF) in Saxony-Anhalt, Germany, was given due to the collaboration with the Helmholtz-Centre for Environmental Research that is oparting the research facility. All samples were obtained and analysed in Germany, and no import / expert was done.

Disturbance | Field work and sample collection activities were conducted with careful consideration to minimize potential disturbances to the ecosystem at the GCEF. Precautions were taken to minimize the use of heavy equipment, thereby reducing the impact on the surrounding environment. Monitoring protocols were implemented to assess any potential disturbances caused by the research activities, and mitigation measures were employed where necessary.

Reporting for specific materials, systems and methods

We require information from authors about some types of materials, experimental systems and methods used in many studies. Here, indicate whether each material, system or method listed is relevant to your study. If you are not sure if a list item applies to your research, read the appropriate section before selecting a response.

Materials & experimental systems

n/a	Involved in the study	
☒	☐	Antibodies
☒	☐	Eukaryotic cell lines
☒	☐	Palaeontology and archaeology
☒	☐	Animals and other organisms
☒	☐	Clinical data
☒	☐	Dual use research of concern
☒	☐	Plants

Methods

n/a	Involved in the study	
☒	☐	ChIP-seq
☒	☐	Flow cytometry
☒	☐	MRI-based neuroimaging

Antibodies

Antibodies used | *Describe all antibodies used in the study; as applicable, provide supplier name, catalog number, clone name, and lot number.*

Validation | *Describe the validation of each primary antibody for the species and application, noting any validation statements on the manufacturer’s website, relevant citations, antibody profiles in online databases, or data provided in the manuscript.*

Eukaryotic cell lines

Policy information about cell lines and Sex and Gender in Research

Cell line source(s) | *State the source of each cell line used and the sex of all primary cell lines and cells derived from human participants or vertebrate models.*

Authentication | *Describe the authentication procedures for each cell line used OR declare that none of the cell lines used were authenticated.*

Mycoplasma contamination | *Confirm that all cell lines tested negative for mycoplasma contamination OR describe the results of the testing for mycoplasma contamination OR declare that the cell lines were not tested for mycoplasma contamination.*

Commonly misidentified lines
(See ICLAC register)

Name any commonly misidentified cell lines used in the study and provide a rationale for their use.

Palaeontology and Archaeology

Specimen provenance

Provide provenance information for specimens and describe permits that were obtained for the work (including the name of the issuing authority, the date of issue, and any identifying information). Permits should encompass collection and, where applicable, export.

Specimen deposition

Indicate where the specimens have been deposited to permit free access by other researchers.

Dating methods

If new dates are provided, describe how they were obtained (e.g. collection, storage, sample pretreatment and measurement), where they were obtained (i.e. lab name), the calibration program and the protocol for quality assurance OR state that no new dates are provided.

☐ Tick this box to confirm that the raw and calibrated dates are available in the paper or in Supplementary Information.

Ethics oversight

Identify the organization(s) that approved or provided guidance on the study protocol, OR state that no ethical approval or guidance was required and explain why not.

Note that full information on the approval of the study protocol must also be provided in the manuscript.

Animals and other research organisms

Policy information about studies involving animals; ARRIVE guidelines recommended for reporting animal research, and Sex and Gender in Research

Laboratory animals

For laboratory animals, report species, strain and age OR state that the study did not involve laboratory animals.

Wild animals

Provide details on animals observed in or captured in the field; report species and age where possible. Describe how animals were caught and transported and what happened to captive animals after the study (if killed, explain why and describe method; if released, say where and when) OR state that the study did not involve wild animals.

Reporting on sex

Indicate if findings apply to only one sex; describe whether sex was considered in study design, methods used for assigning sex. Provide data disaggregated for sex where this information has been collected in the source data as appropriate; provide overall numbers in this Reporting Summary. Please state if this information has not been collected. Report sex-based analyses where performed, justify reasons for lack of sex-based analysis.

Field-collected samples

For laboratory work with field-collected samples, describe all relevant parameters such as housing, maintenance, temperature, photoperiod and end-of-experiment protocol OR state that the study did not involve samples collected from the field.

Ethics oversight

Identify the organization(s) that approved or provided guidance on the study protocol, OR state that no ethical approval or guidance was required and explain why not.

Note that full information on the approval of the study protocol must also be provided in the manuscript.

Clinical data

Policy information about clinical studies

All manuscripts should comply with the ICMJEguidelines for publication of clinical research and a completedCONSORT checklist must be included with all submissions.

Clinical trial registration

Provide the trial registration number from ClinicalTrials.gov or an equivalent agency.

Study protocol

Note where the full trial protocol can be accessed OR if not available, explain why.

Data collection

Describe the settings and locales of data collection, noting the time periods of recruitment and data collection.

Outcomes

Describe how you pre-defined primary and secondary outcome measures and how you assessed these measures.

Dual use research of concern

Policy information about dual use research of concern

Hazards

Could the accidental, deliberate or reckless misuse of agents or technologies generated in the work, or the application of information presented in the manuscript, pose a threat to:

No	Yes	
☒	☐	Public health
☒	☐	National security
☒	☐	Crops and/or livestock
☒	☐	Ecosystems
☒	☐	Any other significant area

Experiments of concern

Does the work involve any of these experiments of concern:

No	Yes	
☒	☐	Demonstrate how to render a vaccine ineffective
☒	☐	Confer resistance to therapeutically useful antibiotics or antiviral agents
☒	☐	Enhance the virulence of a pathogen or render a nonpathogen virulent
☒	☐	Increase transmissibility of a pathogen
☒	☐	Alter the host range of a pathogen
☒	☐	Enable evasion of diagnostic/detection modalities
☒	☐	Enable the weaponization of a biological agent or toxin
☒	☐	Any other potentially harmful combination of experiments and agents

Plants

Seed stocks	*Report on the source of all seed stocks or other plant material used. If applicable, state the seed stock centre and catalogue number. If plant specimens were collected from the field, describe the collection location, date and sampling procedures.*
Novel plant genotypes	*Describe the methods by which all novel plant genotypes were produced. This includes those generated by transgenic approaches, gene editing, chemical/radiation-based mutagenesis and hybridization. For transgenic lines, describe the transformation method, the number of independent lines analyzed and the generation upon which experiments were performed. For gene-edited lines, describe the editor used, the endogenous sequence targeted for editing, the targeting guide RNA sequence (if applicable) and how the editor was applied.*
Authentication	*Describe any authentication procedures for each seed stock used or novel genotype generated. Describe any experiments used to assess the effect of a mutation and, where applicable, how potential secondary effects (e.g. second site T-DNA insertions, mosiacism, off-target gene editing) were examined.*

ChIP-seq

Data deposition

☐ Confirm that both raw and final processed data have been deposited in a public database such as GEO.

☐ Confirm that you have deposited or provided access to graph files (e.g. BED files) for the called peaks.

Data access links<br>*May remain private before publication.*	*For "Initial submission" or "Revised version" documents, provide reviewer access links. For your "Final submission" document, provide a link to the deposited data.*
Files in database submission	*Provide a list of all files available in the database submission.*
Genome browser session<br>(e.g. UCSC)	*Provide a link to an anonymized genome browser session for "Initial submission" and "Revised version" documents only, to enable peer review. Write "no longer applicable" for "Final submission" documents.*

Methodology

Replicates	*Describe the experimental replicates, specifying number, type and replicate agreement.*
Sequencing depth	*Describe the sequencing depth for each experiment, providing the total number of reads, uniquely mapped reads, length of reads and whether they were paired- or single-end.*
Antibodies	*Describe the antibodies used for the ChIP-seq experiments; as applicable, provide supplier name, catalog number, clone name, and lot number.*
Peak calling parameters	*Specify the command line program and parameters used for read mapping and peak calling, including the ChIP, control and index files used.*

Data quality	*Describe the methods used to ensure data quality in full detail, including how many peaks are at FDR 5% and above 5-fold enrichment.*
Software	*Describe the software used to collect and analyze the ChIP-seq data. For custom code that has been deposited into a community repository, provide accession details.*

Flow Cytometry

Plots

Confirm that:

- [ ] The axis labels state the marker and fluorochrome used (e.g. CD4-FITC).
- [ ] The axis scales are clearly visible. Include numbers along axes only for bottom left plot of group (a 'group' is an analysis of identical markers).
- [ ] All plots are contour plots with outliers or pseudocolor plots.
- [ ] A numerical value for number of cells or percentage (with statistics) is provided.

Methodology

Sample preparation	*Describe the sample preparation, detailing the biological source of the cells and any tissue processing steps used.*
Instrument	*Identify the instrument used for data collection, specifying make and model number.*
Software	*Describe the software used to collect and analyze the flow cytometry data. For custom code that has been deposited into a community repository, provide accession details.*
Cell population abundance	*Describe the abundance of the relevant cell populations within post-sort fractions, providing details on the purity of the samples and how it was determined.*
Gating strategy	*Describe the gating strategy used for all relevant experiments, specifying the preliminary FSC/SSC gates of the starting cell population, indicating where boundaries between "positive" and "negative" staining cell populations are defined.*

- [ ] Tick this box to confirm that a figure exemplifying the gating strategy is provided in the Supplementary Information.

Magnetic resonance imaging

Experimental design

Design type	*Indicate task or resting state; event-related or block design.*
Design specifications	*Specify the number of blocks, trials or experimental units per session and/or subject, and specify the length of each trial or block (if trials are blocked) and interval between trials.*
Behavioral performance measures	*State number and/or type of variables recorded (e.g. correct button press, response time) and what statistics were used to establish that the subjects were performing the task as expected (e.g. mean, range, and/or standard deviation across subjects).*

Acquisition

Imaging type(s)	*Specify: functional, structural, diffusion, perfusion.*
Field strength	*Specify in Tesla*
Sequence & imaging parameters	*Specify the pulse sequence type (gradient echo, spin echo, etc.), imaging type (EPI, spiral, etc.), field of view, matrix size, slice thickness, orientation and TE/TR/flip angle.*
Area of acquisition	*State whether a whole brain scan was used OR define the area of acquisition, describing how the region was determined.*

Diffusion MRI ☐ Used ☐ Not used

Preprocessing

Preprocessing software	*Provide detail on software version and revision number and on specific parameters (model/functions, brain extraction, segmentation, smoothing kernel size, etc.).*
Normalization	*If data were normalized/standardized, describe the approach(es): specify linear or non-linear and define image types used for transformation OR indicate that data were not normalized and explain rationale for lack of normalization.*

Normalization template | *Describe the template used for normalization/transformation, specifying subject space or group standardized space (e.g. original Talairach, MNI305, ICBM152) OR indicate that the data were not normalized.*

Noise and artifact removal | *Describe your procedure(s) for artifact and structured noise removal, specifying motion parameters, tissue signals and physiological signals (heart rate, respiration).*

Volume censoring | *Define your software and/or method and criteria for volume censoring, and state the extent of such censoring.*

Statistical modeling & inference

Model type and settings | *Specify type (mass univariate, multivariate, RSA, predictive, etc.) and describe essential details of the model at the first and second levels (e.g. fixed, random or mixed effects; drift or auto-correlation).*

Effect(s) tested | *Define precise effect in terms of the task or stimulus conditions instead of psychological concepts and indicate whether ANOVA or factorial designs were used.*

Specify type of analysis: ☐ Whole brain ☐ ROI-based ☐ Both

Statistic type for inference | *Specify voxel-wise or cluster-wise and report all relevant parameters for cluster-wise methods.*

(See Eklund et al. 2016)

Correction | *Describe the type of correction and how it is obtained for multiple comparisons (e.g. FWE, FDR, permutation or Monte Carlo).*

Models & analysis

n/a	Involved in the study
☐	☐ Functional and/or effective connectivity
☐	☐ Graph analysis
☐	☐ Multivariate modeling or predictive analysis

Functional and/or effective connectivity | *Report the measures of dependence used and the model details (e.g. Pearson correlation, partial correlation, mutual information).*

Graph analysis | *Report the dependent variable and connectivity measure, specifying weighted graph or binarized graph, subject- or group-level, and the global and/or node summaries used (e.g. clustering coefficient, efficiency, etc.).*

Multivariate modeling and predictive analysis | *Specify independent variables, features extraction and dimension reduction, model, training and evaluation metrics.*
