# Supplementary material for: Sustainable land management enhances ecological and economic multifunctionality under ambient and future climate
Source: Nat Commun. 2024 Jun 10;15:4930. doi: 10.1038/s41467-024-48830-z (PMC11164979; doi:10.1038/s41467-024-48830-z)
Supplement: Supplementary file 3 — Description of Additional Supplementary Files [file 41467_2024_48830_MOESM3_ESM.pdf]

File Name: Supplementary Data 1

Description: Pairwise comparisons of estimated marginal means (EMMs) for the effects of LUT (land use type), climate, and the land use-climate interaction on farmers' ecological multifunctionality). The table presents the contrasts, estimates, standard errors (SE), degrees of freedom (df), t-ratios, and p-values. Significant differences between LUT categories are determined using the Tukey adjustment method, whereas bold values indicate a significant effect of the respective factor or interaction (*** p<0.001; ** p<0.01; * p<0.05). Negative estimates indicate a decrease in farmers' ecological multifunctionality compared to the reference category. Positive estimates indicate an increase in farmers ' ecological multifunctionality compared to the reference category. In the context of the Tukey test, the unexpected degrees of freedom observed in the results can be attributed to the adjustment made to account for unequal variances among the groups or combinations being compared. This adjustment ensures the validity of the pairwise comparisons by accurately estimating the standard errors and adjusting the degrees of freedom to accommodate the variability in variances across the data.

File Name: Supplementary Data 2

Description: Pairwise comparisons of estimated marginal means (EMMs) for the effects of LUT (land use type), climate, and the land use-climate interaction on local residents' ecological multifunctionality. The table presents the contrasts, estimates, standard errors (SE), degrees of freedom (df), t-ratios, and p-values. Significant differences between LUT categories are determined using the Tukey adjustment method, whereas bold values indicate a significant effect of the respective factor or interaction (*** p<0.001; ** p<0.01; * p<0.05). Negative estimates indicate a decrease in local residents' ecological multifunctionality compared to the reference category. Positive estimates indicate an increase in local residents' ecological multifunctionality compared to the reference category. In the context of the Tukey test, the unexpected degrees of freedom observed in the results can be attributed to the adjustment made to account for unequal variances among the groups or combinations being compared. This adjustment ensures the validity of the pairwise comparisons by accurately estimating the standard errors and adjusting the degrees of freedom to accommodate the variability in variances across the data.

File Name: Supporting Data File 3

Description: Pairwise comparisons of estimated marginal means (EMMs) for the effects of LUT (land use type), climate, and the land use-climate interaction on tourism sector's ecological multifunctionality. The table presents the contrasts, estimates, standard errors (SE), degrees of freedom (df), t-ratios, and p-values. Significant differences between LUT categories are determined using the Tukey adjustment method, whereas bold values indicate a significant effect of the respective factor or interaction (*** p<0.001; ** p<0.01; * p<0.05). Negative estimates indicate a decrease in tourism sector's ecological multifunctionality compared to the reference category. Positive estimates indicate an increase in tourism sector's ecological multifunctionality compared to the reference category. In the context of the Tukey test, the unexpected degrees of freedom observed in the results can be attributed to the adjustment made to account for unequal variances among the groups or combinations being compared. This adjustment ensures the validity of the pairwise comparisons by accurately estimating the standard errors and adjusting the degrees of freedom to accommodate the variability in variances across the data.

File Name: Supporting Data File 4

Description: Correlation matrix showing the pairwise correlations and p-values among selected variables in the study. The correlation values range from -1 to 1, representing the strength and direction of the relationships between the variables. The corresponding p-values indicate the statistical significance of the correlations, with lower values suggesting stronger evidence of a significant relationship (*** $p<0.001$; ** $p<0.01$; * $p<0.05$).

File Name: Supporting Data File 5

Description: Transfer of the weighting factors. Relative ecosystem service preferences of the four different stakeholder groups most relevant for this study as obtained from Peter et al. (2021) and overview of the process to transfer the preferences to the ecosystem services used within this study. Ecosystem services relevant for this study are marked in grey. For the purpose of this study, a new ecosystem service preference ‘food production’ was calculated for each stakeholder group as the mean of the two ecosystem services ‘food production (from crops)’ and ‘livestock production’ (marked with asterisks). † Classification of ecosystem service categories are based on Millenium Ecosystem Assessment (MEA, 2005) and can deviate from the classification used in Peter et al. (2021). E.g., the ecosystem service ‘biodiversity’ was classified as cultural service in Peter et al. (2021) and was classified as supporting service for the context of this study.
